# Prediction accuracy of conventional and total keratometry for intraocular lens power calculation in femtosecond laser-assisted cataract surgery

**DOI:** 10.1038/s41598-021-92354-1

**Published:** 2021-06-18

**Authors:** Soyoung Ryu, Ikhyun Jun, Tae-im Kim, Kyoung Yul Seo, Eung Kweon Kim

**Affiliations:** 1 Department of Ophthalmology, The Institute of Vision Research, Yonsei University College of Medicine, 50-1 Yonsei-ro, Seodaemungu, Seoul, 03722 Republic of Korea; 2 Department of Ophthalmology, Corneal Dystrophy Research Institute, Yonsei University College of Medicine, Seoul, Republic of Korea; 3 Saevit Eye Hospital, Goyang-Si, Gyeonggi-Do Republic of Korea

**Keywords:** Eye diseases, Medical imaging

## Abstract

This study evaluated the accuracy of total keratometry (TK) and standard keratometry (K) for intraocular lens (IOL) power calculation in eyes treated with femtosecond laser-assisted cataract surgery. The retrospective study included a retrospective analysis of data from 62 patients (91 eyes) who underwent uneventful femtosecond laser-assisted cataract surgery with Artis PL E (Cristalens Industrie, Lannion, France) IOL implantation by a single surgeon between May 2020 and December 2020 in Severance Hospital, Seoul, South Korea. The new IOLMaster 700 biometry device (Carl Zeiss Meditec, Jena, Germany) was used to calculate TK and K. The mean absolute error (MAE), median absolute error (MedAE), and the percentages of eyes within prediction errors of ± 0.25 D, ± 0.50 D, and ± 1.00 D were calculated for all IOL formulas (SRK/T, Hoffer-Q, Haigis, Holladay 1, Holladay 2, and Barrett Universal II). There was strong agreement between K and TK (intraclass correlation coefficient = 0.99), with a mean difference of 0.04 D. For all formulas, MAE tended to be lower for TK than for K, and relatively lower MAE and MedAE values were observed for SRK/T and Holladay 1. Furthermore, for all formulas, a greater proportion of eyes fell within ± 0.25 D of the predicted postoperative spherical equivalent range in the TK group than in the K group. However, differences in MAEs, MedAEs, and percentages of eyes within the above prediction errors were not statistically significant. In conclusion, TK and K exhibit comparable performance for refractive prediction in eyes undergoing femtosecond laser-assisted cataract surgery.

## Introduction

Patient satisfaction after cataract surgery is largely dependent upon precise predictions of refractive outcomes, highlighting the importance of rapid technological advancements in methods for measuring ocular parameters and intraocular lens (IOL) power. Among the keys to accurate prediction of IOL power is precise measurement of the keratometric value. Today, standard keratometry (K) relies purely on measurements of the anterior corneal surface, and even when the posterior corneal surface is taken into consideration, the refractive index of the posterior corneal surface has been mostly inferred by calculation based on model eyes or nomograms^1^. Therefore, recent studies have debated the addition of accurate posterior corneal measurements to formulas for calculating IOL in patients undergoing cataract surgery^2,3^.

Recently, a new biometry device known as the IOLMaster 700 (Carl Zeiss Meditec, Jena, Germany), based on the principle of swept-source optical coherence tomography (SS-OCT), has been developed. IOLMaster 700 enables the assessment of the posterior corneal surface by combining data from the anterior corneal surface obtained via telecentric keratometry with pachymetry data obtained via SS-OCT^4–7^. Subsequently, total keratometry (TK) values are calculated using data from both the anterior and posterior cornea and measurements of corneal thickness, which are combined using the thick lens formula^4^.

Some studies have reported better refractive outcomes for conventional monofocal IOL implantation when accurate posterior corneal data are used to calculate TK values, whereas few other studies did not show any benefits of TK over K^2,4,8–10^. A recent study also compared refractive outcomes of cataract surgery with diffractive multifocal IOLs using K and TK, and K groups showed better IOL power prediction accuracy than TK groups across most of the formulas (Haigis, Holladay2, Barrett Universal II), except for the SRK/T^11^. Lawless et al. reported that the Barrett True-K using TK showed the lowest prediction error in eyes with previous laser refractive surgery^12^. Favorable results have also been documented for calculations associated with toric IOL implantation, which is more often expected to reduce postoperative astigmatism^3,13,14^. Similar to toric IOLs, femtosecond laser-assisted astigmatic keratotomy during femtosecond laser-assisted cataract surgery is among the preferred solutions for the correction of preoperative astigmatism. Therefore, accurate measurements of keratometry and subsequent optimal IOL power selection are crucial to ensure favorable postoperative refractive results. However, there is a paucity of data evaluating refractive outcomes using TK values in patients undergoing femtosecond laser-assisted cataract surgery. Femtosecond laser-assisted cataract surgery is a new technology which was introduced approximately half a decade ago. With potential benefits of more accurate capsulotomy size, shape, and positioning, and less IOL tilt with fewer higher-order aberrations, many surgeons integrate laser technology into their practice over conventional cataract surgery procedure^15–17^. Therefore, with the growing popularity, there is a need to evaluate the benefit of using TK for IOL power calculation in patients undergoing femtosecond laser-assisted cataract surgery.

Therefore, the present study aimed to evaluate the agreement of K and TK values measured using IOLMaster 700 in eyes undergoing femtosecond laser-assisted cataract surgery. Subsequently, the study compared the accuracy of K and TK in predicting residual refraction after femtosecond laser-assisted cataract surgery using existing standard formulas, including SRK/T, Hoffer-Q, Haigis, Holladay 1, Holladay 2, and Barrett Universal II.

## Results

### Patient characteristics and preoperative measurements

Patient characteristics and ocular biometry measurements, including axial length (AL), anterior chamber depth (ACD), lens thickness (LT), white-to-white (WTW), corneal center thickness (CCT), obtained using IOLMaster 700 are shown in Table 1. A total of 91 eyes (46 right, 45 left) of 62 patients (32 men, 30 women) with a mean age of 68.01 ± 10.07 years were included for the analysis. The mean AL was 23.89 ± 1.76 mm, and the mean ACD was 3.14 ± 0.40 mm. The mean magnitudes of preoperative K and TK values measured using IOLMaster 700 were 44.20 ± 1.46 D and 44.24 ± 1.48 D, respectively, while the corresponding median K and TK values were 44.22 D and 44.25 D, respectively. The mean preoperative and postoperative best-corrected visual acuity (BCVA) (LogMAR) were 0.39 ± 0.26 and 0.06 ± 0.22, respectively. The mean preoperative and postoperative SE was − 1.55 ± 4.78 and -0.48 ± 0.25, respectively. The mean IOL power selected by the surgeon was 21.18 ± 4.14 D.

**Table 1:** Patient characteristics and ocular biometry data.

Parameter	
Patients/eyes	62/91
Right/left	46/45
Male/female	32/30
Age (y)	68.01 ± 10.07
Preop BCVA (LogMAR)	0.39 ± 0.26
Postop BCVA (LogMAR)	0.06 ± 0.22
AL (mm)	23.89 ± 1.76
ACD (mm)	3.14 ± 0.40
LT (mm)	4.46 ± 0.47
WTW (mm)	11.76 ± 0.47
CCT (μm)	538.53 ± 31.92
Keratometry (D)	44.20 ± 1.46
Total keratometry (D)	44.24 ± 1.48
IOL SE power	21.18 ± 4.14
Preop refraction (SE)	− 1.55 ± 4.78
Postop refraction (SE)	− 0.48 ± 0.25
Preop keratometry (D) (Scheimpflug)	44.14 ± 1.57
Postop keratometry (D) (Scheimpflug)	44.23 ± 1.58
Preop corneal astigmatism (D)	0.95 ± 0.60
Postop corneal astigmatism (D)	0.80 ± 0.46

Results are expressed as means ± standard deviation.

*BCVA* best-corrected visual acuity, *AL* axial length, *ACD* anterior chamber depth, *LT* lens thickness, *WTW* white-to-white distance, *CCT* corneal center thickness, *IOL* intraocular lens, *SE* spherical equivalent.

### Effect of femtosecond laser-assisted keratotomy on keratometric values

The mean magnitudes of preoperative and postoperative K values measured using Scheimpflug-based corneal topography were 44.14 ± 1.57 D and 44.23 ± 1.58 D, respectively. There was no significant difference between preoperative and postoperative keratometric values (*P* = 0.296). The mean magnitudes of preoperative and postoperative corneal astigmatism values measured using Scheimpflug-based corneal topography were 0.95 ± 0.60 D and 0.80 ± 0.46 D, respectively. There was a significant difference between preoperative and postoperative corneal astigmatism (*P* = 0.005).

### Agreement between K and TK

**Figure 1:**
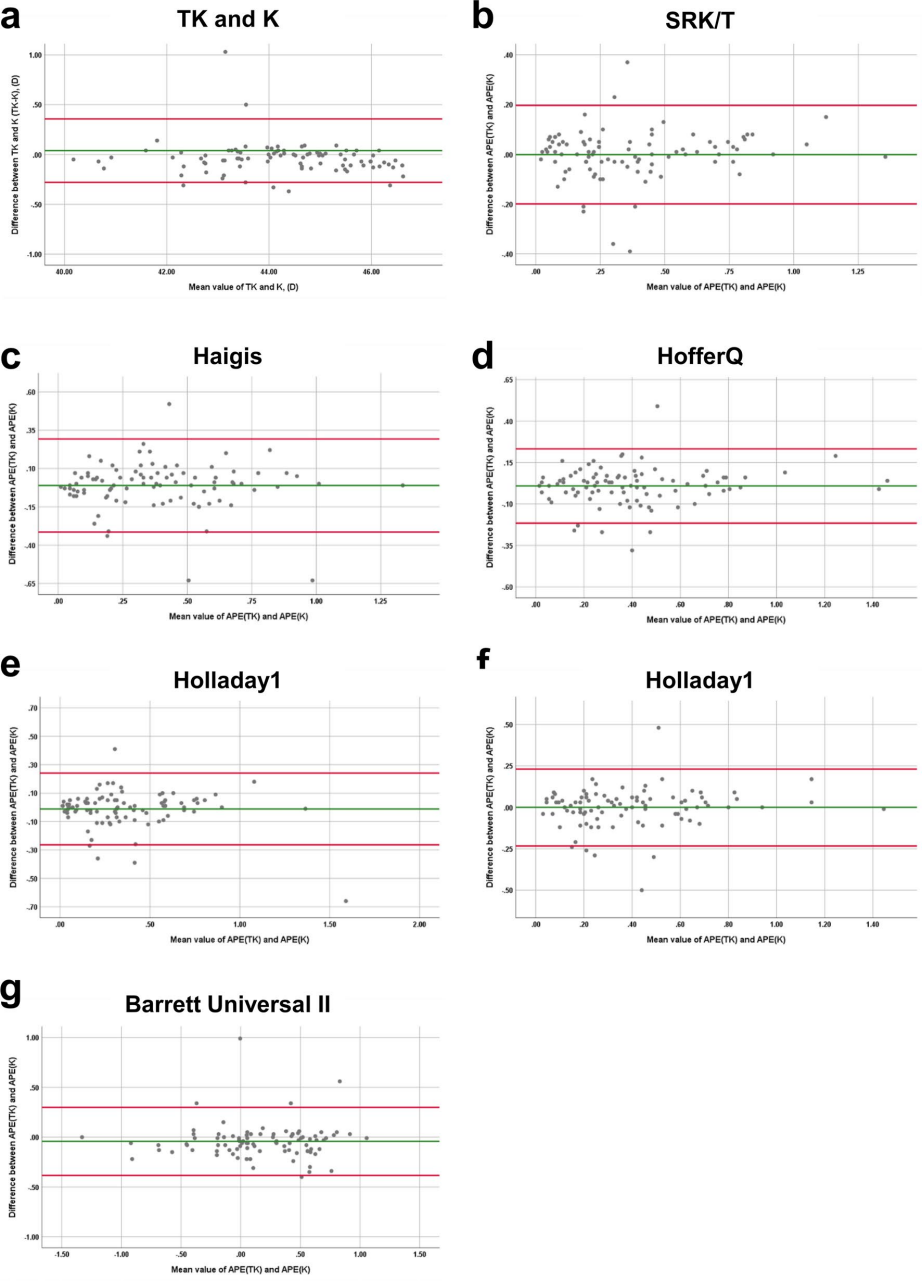
Agreement between conventional keratometry (K) and total keratometry (TK) for the normal range of keratometry values. A Bland–Altman plot between standard keratometry (K) and total keratometry (TK). (**b**–**g**) Agreement between absolute prediction error (APE) of spherical equivalent (SE) from standard keratometry (K) and total keratometry (TK) values. A Bland–Altman plot showing (**b**) APE of SE calculated using the SRK/T formula. (**c**) APE of SE calculated using the Haigis formula. (**d**) APE of SE calculated using the Hoffer-Q formula. (**e**) APE of SE calculated using the Holladay 1 formula. (**f**) APE of SE calculated using the Holladay 2 formula. (**g**) APE of SE calculated using the Barrett Universal II formula.

**Figure 2:**
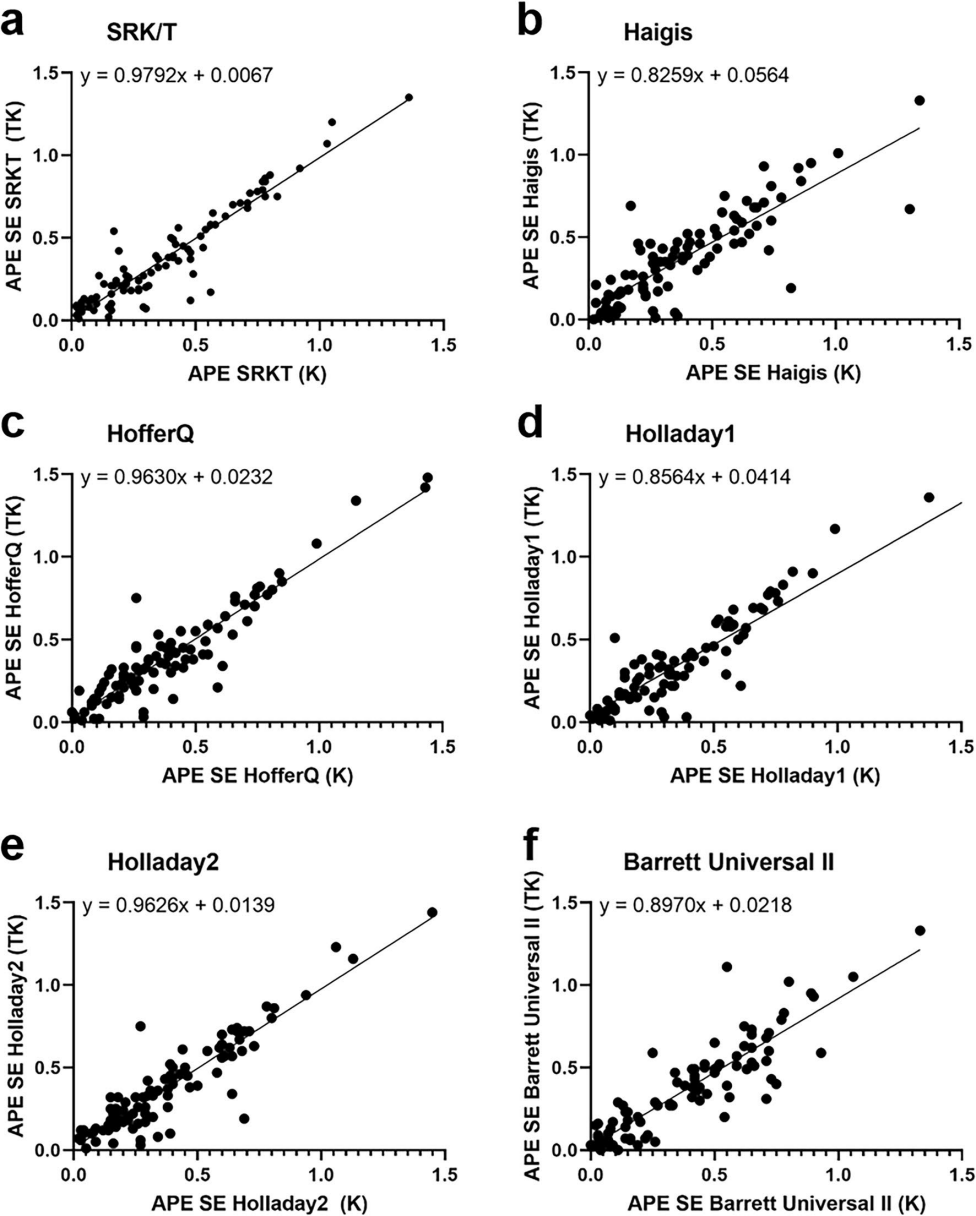
Pairwise comparison of absolute prediction error (APE) of spherical equivalent (SE) from standard keratometry (K) and total keratometry (TK) values. (**a**) APE of SE calculated using the SRK/T formula. (**b**) APE of SE calculated using the Haigis formula. (**c**) APE of SE calculated using the Hoffer-Q formula. (**d**) APE of SE calculated using the Holladay 1 formula. (**e**) APE of SE calculated using the Holladay 2 formula. (**f**) APE of SE calculated using the Barrett Universal II formula.

The mean difference between K and TK (K–TK) was 0.04 D, and the ICC between the two groups was 0.99, indicative of very good agreement. As shown in Fig. 1a, the Bland–Altman plot of K and TK also demonstrates a fine agreement between the two groups. In addition, the Bland–Altman plot and pairwise comparisons of absolute prediction errors (APE) values for K and TK obtained using multiple IOL formulas are shown in Figs. 1b–g and 2.

### Refractive outcomes

Refractive errors, mean absolute errors (MAEs) and median absolute errors (MedAEs), calculated using six standard formulas (SRK/T, Hoffer-Q, Haigis, Holladay 1, Holladay 2, and Barrett Universal II) based on optimized IOL constants are shown in Table 2. The MAEs ranged from 0.370 to 0.397 D in the K group and from 0.358 to 0.404 D in the TK group. Although the MAEs tended to be lower in the TK group than in the K group for all formulas (except Hoffer-Q), the magnitudes of differences were small and not statistically significant (Wilcoxon signed-rank tests, all *P* values > 0.05). Additionally, the MAE and MedAE values were relatively lower for the SRK/T and Holladay 1 formulas than for other formulas; however, there were no significant differences between formulas (Friedman test with Bonferroni adjustment, *P* = 0.202).

**Table 2:** Mean absolute errors (MAEs) and median absolute errors (MedAEs) for all formulas using keratometry (K) and total keratometry (TK) values.

IOL formula	K Group					TK Group					P value between K and TK
	MAE	SD	MedAE	Min	Max	MAE	SD	MedAE	Min	Max	
SRK/T	0.37	0.28	0.30	0.02	1.36	0.37	0.29	0.27	0.01	1.35	0.56
Haigis	0.39	0.28	0.33	0.02	1.34	0.38	0.27	0.36	0.00	1.33	0.93
HofferQ	0.40	0.29	0.33	0.00	1.44	0.40	0.30	0.34	0.01	1.48	0.18
Holladay1	0.37	0.31	0.30	0.00	1.92	0.36	0.29	0.29	0.01	1.36	0.99
Holladay2	0.39	0.27	0.32	0.02	1.45	0.39	0.28	0.33	0.01	1.44	0.46
Barrett II	0.40	0.28	0.41	0.00	1.33	0.38	0.29	0.36	0.00	1.33	0.20

Wilcoxon signed-rank test for mean difference, all *p* values > 0.05 between the K and TK groups. Friedman test with Bonferroni post-hoc correction for multiple comparisons between IOL formulas, *p* value = .977.

*IOL* intraocular lens, *K* keratometry, *TK* total keratometry.

**Figure 3:**
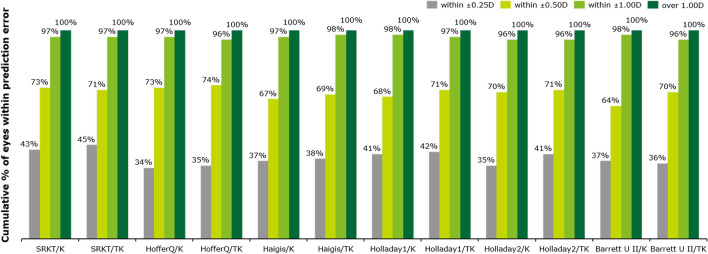
Stacked histogram comparing the percentages of eyes within ± 0.25 D, ± 0.50 D, and ± 1.00 D of predicted postoperative spherical equivalent refraction (SE) between all formulas using keratometry (K) and total keratometry (TK).

The proportions of eyes within ± 0.25 D, ± 0.5 D, and ± 1.00 D of predicted postoperative spherical equivalent (SE) across all formulas are shown in Fig. 3 and Table 3. The proportion of eyes within ± 0.25 D of the predicted range for postoperative SE was higher in the TK group than in the K group for all formulas except for the Barrett Universal II, and a similar trend was observed for eyes falling within the ± 0.5 D range for the majority of formulas (Haigis, Hoffer-Q, Holladay 1, Holladay 2, Barrett Universal II). However, there were no significant differences between the groups (McNemar’s chi-square test, all *P* values > 0.05).

**Table 3:** Percentage of eyes within ± 0.25 D, ± 0.50 D, and ± 1.00 D of predicted postoperative spherical equivalent refraction (SE) between all formulas using K and TK.

IOL formula	Postoperative spherical equivalent refraction (D)					
	± 0.25D		± 0.50D		± 1.00D	
	K	TK	K	TK	K	TK
SRK/T	42.9	45.1	72.5	71.4	96.7	96.7
Haigis	36.3	37.4	68.1	70.3	96.7	97.8
HofferQ	33.0	33.0	71.4	73.6	96.7	95.6
Holladay1	40.7	40.7	68.1	71.4	97.8	96.7
Holladay2	34.1	38.5	70.3	69.2	95.6	95.6
Barrett II	36.3	36.3	61.5	69.2	97.8	95.6

Paired McNemar’s chi-square test, all *p* values > 0.05 between the K and TK groups.

*K* keratometry, *TK* total keratometry, *IOL* intraocular lens.

## Discussion

Today, standard K relies purely on measurements of the anterior corneal surface, and the effect of posterior curvature is only predicted based on mathematical extrapolation using nomograms, such as the Baylor nomogram or Barrett Toric calculator. Due to its nature, the possibility of incorrect estimation of total corneal astigmatism remains a concern.

Therefore, several new technologies enabling direct measurement of posterior corneal curvature have been developed, leading to the introduction of the concept of “total corneal power.” However, depending on the device used to measure posterior corneal astigmatism, the use of total corneal power has been associated with varying outcomes in patients undergoing uncomplicated cataract surgery^18^. In one study, total corneal power, which was measured using topography from a Scheimpflug camera system, was less accurate in predicting IOL power than conventional K^9^. However, recent studies have demonstrated improvements in refractive outcomes using “total keratometry” values derived from IOLMaster 700, an SS-OCT-type optical biometry device for IOL calculation. Srivannaboon et al.^4^ reported strong agreement between K and TK, with a trend toward better refractive outcomes using TK in conventional cataract surgery^4^. However, other studies which investigated the patients undergoing multi-focal IOL implantation and the patients who underwent cataract surgery after myopic laser in situ keratomileusis did not find critical refractive benefit of TK over K in IOL power calculation^10,11^. Until now, few studies have aimed to validate the benefits of using TK values derived via SS-OCT-type optical biometry, and no studies assessing the compatibility of TK with IOL calculations in femtosecond laser-assisted cataract surgery have been published. Therefore, we aimed to evaluate the benefit of using TK for IOL power calculation in patients undergoing femtosecond laser-assisted cataract surgery. The analysis of this study indicated that TK and K exhibited comparable performance for refractive prediction in eyes undergoing the procedure.

This study follows the method for IOL power studies recommended by Hoffer et al.^19^ except that the assessment of accuracy was based on the MAE, as suggested by Kane et al.^20^ Using a single IOL model, a single surgeon performed all surgeries with an optimized IOL constant to minimize the potential bias from variations in operating styles or techniques^21^.

Previous studies have reported higher prediction accuracy for IOL calculation in patients undergoing conventional cataract surgery using TK than using K, with strong agreement between K and TK values^2,4^. Fabian et al. reported that the MAEs in SE within ± 0.5 D calculated using the Barrett Universal II formula were 84% (K group) versus 86% (TK group) in a post hoc analysis including 145 eyes^2^. Srivannaboon et al. also reported a trend toward lower MAEs and MedAEs for TK when compared with K when using the SRK/T, Hoffer-Q, Haigis, Holladay 1 and 2, and Barrett and Barrett TK Universal formulas. Moreover, they observed strong agreement between K and TK values, with a mean difference of 0.03 D between K and TK^4^. Despite the difference in the surgical procedure, our results are very similar to those of previous studies, supporting the compatibility of TK with IOL calculation.

We observed a strong agreement between TK and K, and there was a trend toward better refractive outcomes in the TK group than in the K group, considering lower MAE values and bigger percentages of eyes within the above prediction errors. This was not surprising because the conventional K values are based on mathematical extrapolation of the posterior corneal curvature and thus cannot take outliers and irregularities into account^2^. However, the magnitudes of the differences were small between the two groups and not statistically different. Therefore, although we have demonstrated comparable performance of TK in refractive prediction in eyes undergoing femtosecond laser-assisted cataract surgery, further studies need to be done whether there is superiority of TK over K values. It is unexpected that the Barrett Universal II formula yielded the lowest percentage of eyes (61.5% and 69.2%, respectively) with a prediction of ± 0.5 D in both the TK and K groups when compared with other formulas, given that recent studies have demonstrated the Barrett Universal II formula to have the lowest absolute error when compared with other modern formulas^20,22,23^. However, Srivannaboon et al., who investigated refractive outcomes using TK and K for IOL power calculation in conventional cataract cases, also reported that the Barrett formula yielded the lowest percentage of eyes within the specified range (K: 59.6%, TK: 65.4%). Srivannaboon et al. pointed out that this may be due to the use of optimized IOL constants instead of personalized IOL constants^4^. In addition, the effect of astigmatic changes after femtosecond laser-assisted astigmatic keratotomy may have played a role in our patients. And it may be associated with the shape of IOL of this study. The IOL used in the current study is slightly small sized with 10.50 mm ~ 11.00 mm overall diameter, and 5.80 mm ~ 6.15 mm optic diameter, and has four closed loop haptic. Further studies with a larger number of patients in each group should be conducted to verify our results.

This study has some limitations. First, the study was confined to patients with biometric parameters falling within the normal range. Therefore, these results cannot be applied to patients with extreme parameters, such as long/short ALs, post-refractive surgery eyes, cases of keratoconus, and others. Second, we did not consider the effect of femtosecond laser-assisted astigmatic keratotomy. Personalized laser keratotomy settings may have introduced bias in our results. However, previous studies have reported no significant difference in the mean postoperative SE between the femtosecond laser-assisted cataract and manual phacoemulsification groups^24^. Due to a lack of post-operative IOLMaster 700 measurements, the mean corneal front K values measured via Scheimpflug-based corneal topography were compared before and after cataract surgery, and no statistically significant difference was noted (paired sample *t*-test, *P* value = 0.296). Therefore, we assumed that the additional step of femtosecond laser-assisted astigmatic keratotomy would not have changed our results to a great extent.

In conclusion, our preliminary results suggest that TK and K values exhibit comparable performance in IOL calculations for femtosecond laser-assisted cataract surgery when the same optimized IOL constant is used, as has been observed in conventional cataract surgery. As this is the first study to investigate the applicability of TK in femtosecond laser-assisted cataract surgery using multiple current standard IOL formulas, further studies involving more eyes and different IOL models are necessary to elucidate the benefit of TK in IOL calculation.

## Methods

### Participants

This retrospective, observational case series was approved by the Institutional Review Board (IRB) of the Yonsei University College of Medicine (4-2021-0131) and was conducted in accordance with the tenets outlined in the Declaration of Helsinki. Informed consent was waived by the IRB of Yonsei University College of Medicine due to the retrospective nature of the study. The study included 91 eyes of 62 patients who had undergone uneventful femtosecond laser-assisted cataract surgery by a single surgeon at Severance Hospital in Seoul, Korea, between May 2020 and December 2020.

Inclusion criteria were as follows: availability of preoperative TK data from IOLMaster 700, auto-refraction performed at 3 months after cataract surgery, absence of complications during or after cataract surgery, and Artis PL E (Cristalens industrie, Lannion, France) IOL implantation during surgery. Patients who had undergone previous ocular surgery; those who had experienced ocular trauma, ocular diseases, or opacities that may impair visual acuity; those with active ocular infection or inflammation; and those who failed to keep postoperative follow-up appointments were excluded from the study.

### Preoperative and postoperative assessments

All patients underwent a comprehensive preoperative ophthalmologic examination performed within 3 months before cataract surgery. This included assessments of BCVA, intraocular pressure (IOP) (CANON, TX-20, Japan), manifest refraction, keratometry, auto-refraction, slit-lamp examination, fundoscopy, retinal examination using Heidelberg spectralis optical coherence tomography (software V.5.4.7.0; Heidelberg Engineering, Heidelberg, Germany), specular microscopy (Tomey, EM4000, GmbH, Germany), and Scheimpflug-based corneal topography (Pentacam HR, Oculus, Wetzlar, Germany). All optical biometric parameters, including K, TK, AL, CCT, LT, WTW, and ACD, were measured using IOLMaster 700. A single experienced surgeon selected the IOL power for each patient according to surgical preferences, and predicted postoperative spherical equivalent refractions for all formulas were documented. The patients were examined 1 day, 1 week, 1 month, and 3 months postoperatively. Slit-lamp examination, IOP measurements, K, auto-refraction assessments, manifest refraction assessments, and corneal topography were performed at each follow-up visit.

### Surgical technique

All patients underwent scheduled cataract surgery, which was performed by a single, experienced surgeon (I.J.). Femtosecond laser-assisted cataract surgery was performed using the LenSx platform (Alcon Laboratories, Inc., Fort Worth, TX, USA) for capsulorhexis, nucleus fragmentation, and penetrating arcuate keratotomy. The keratotomies were centered on the steep corneal axis, and the length of the arcuate keratotomy was determined using a Verion system (Alcon Laboratories, Inc.). After all pattern selections and parameter choices were made, patients were instructed to lie on a built-in bed beneath the laser device. The disposable vacuum interface was positioned to the operation eye with a suction ring, and laser emission was initiated, following which the patient was transported to the operation room. At the start of cataract surgery, laser corneal incision sites were carefully dissected using a Sinskey hook. The incised anterior capsule button was removed using forceps. Patients underwent conventional phacoemulsification using a Centurion Vision System (Alcon Laboratories, Inc.). Following phacoemulsification, a single type of IOL, Artis PL E, was implanted in the capsular bag using an injector, and the remaining ophthalmic visco-surgical device was removed. All incision sites were hydrated to prevent leakage.

### Statistical analyses

Data were analyzed using SPSS Statistics software (version 25; IBM Corporation, Armonk, NY) and Microsoft Excel 2016 (Microsoft Corp, USA). *P* values < 0.05 were considered statistically significant. Descriptive statistics including standard deviations, means, medians, and frequencies were determined.

The APEs, MAEs, MedAEs, and distributions of eyes within ± 0.25 D, ± 0.5 D, and ± 1.00 D of predicted postoperative SE refraction were calculated. The APE of SE was defined as the absolute difference between the actual postoperative SE and predicted postoperative SE. Similarly, the MAE of SE was defined as the mean absolute difference between the actual postoperative SE and predicted postoperative SE, while the MedAE of SE was defined as the median absolute difference between the actual postoperative SE and predicted postoperative SE^25^.

The percentages of eyes within ± 0.25 D, ± 0.5 D, and ± 1.00 D of predicted postoperative SE in the K and TK groups were compared using paired McNemar’s chi-square tests. The differences between the APEs obtained from the K and TK data using each formula was compared using Wilcoxon signed-rank tests. The APEs from the K and TK data of all formulas were compared using the Friedman test for multiple comparisons. Agreement between K and TK was assessed using the Bland–Altman plot method, and intraclass correlation coefficients (ICCs) with 95% confidence intervals were calculated to compare K and TK values^26^.
